# 5T Non-Contrast One-Stop MRA for Thoracic Aorta and Coronary Arteries: A Prospective Feasibility Study in Healthy Volunteers

**DOI:** 10.3390/jcdd13100485

**Published:** 2026-09-26

**Authors:** Yan Xu, Chuangwei Wei, Runzhi Zhang, Jiahuan Dai, Hui Xu, Ke Xue, Nan Zhang, Jiayi Liu, Lei Xu, Zhaoying Wen

**Affiliations:** 1 Department of Radiology, Beijing Anzhen Hospital, Capital Medical University, Beijing 100029, China; xuyan4458@163.com (Y.X.);; 2 Magnetic Resonance Research Collaboration, Beijing United Imaging Research Institute of Intelligent Imaging, Beijing 100094, China; 3 Department of Magnetic Resonance Imaging, Qinghai Provincial People’s Hospital, Xining 810007, China; 4 Magnetic Resonance Research Collaboration, Shanghai United Imaging Healthcare Co., Ltd., Shanghai 201807, China

**Keywords:** 5T, non-contrast MRA, one-stop MRI, thoracic aorta, coronary artery

## Abstract

Background/Objectives: This prospective study evaluated the feasibility of a non-contrast 5T one-stop MRA protocol for simultaneous assessment of the thoracic aorta and coronary arteries and examined the effect of heart rate (HR) on image quality. Methods: Thirty-four healthy volunteers underwent 5T MRA and were stratified by median HR into Low-HR (≤70 bpm, *n* = 17) and High-HR (>70 bpm, *n* = 17) groups. Subjective image quality was assessed using a 4-point scale, and signal-to-noise ratio (SNR), contrast-to-noise ratio (CNR), and vessel edge sharpness (VES) were measured in predefined aortic and proximal coronary segments. Results: The protocol was successfully completed in all participants, and all evaluated segments were evaluable. Interobserver and intraobserver agreement was excellent (kappa ≥ 0.90; ICC ≥ 0.85). Subjective aortic scores did not differ between HR groups, whereas several coronary segments had lower scores in the High-HR group. SNR, CNR, and VES were higher in the Low-HR group across all objectively assessed segments. Conclusions: These findings support the technical feasibility of integrated 5T non-contrast MRA in healthy volunteers and suggest that the subjective effect of higher HR is more evident in the coronary arteries than in the thoracic aorta, warranting further validation in patients with cardiovascular disease.

## 1. Introduction

Comprehensive assessment of the thoracic aorta and coronary arteries is clinically relevant in several cardiovascular settings, particularly when both aortic anatomy and coronary status may influence patient management. Computed tomography angiography (CTA) provides rapid, high-spatial-resolution evaluation of these vascular territories and remains widely used in clinical practice [1,2]. However, exposure to ionizing radiation and the administration of iodinated contrast agents may be of concern in patients requiring repeated examinations or in those for whom iodinated contrast use is undesirable. In this context, non-contrast magnetic resonance angiography (MRA) provides a radiation-free and contrast-agent-free approach that may be particularly useful for longitudinal cardiovascular assessment [3,4,5].

Simultaneous visualization of the thoracic aorta and coronary arteries within a single MRA acquisition is technically demanding because the two vascular territories have substantially different imaging requirements. Thoracic aortic imaging requires broad anatomical coverage and relatively homogeneous signal over a large field of view, whereas coronary MRA requires high spatial resolution and effective suppression of cardiac and respiratory motion because of the small caliber and complex motion of the coronary arteries [6,7]. Previous studies have demonstrated the feasibility of free-breathing, non-contrast three-dimensional MRA for thoracic aortic assessment [4,5], while whole-heart approaches have enabled comprehensive three-dimensional visualization of cardiac and thoracic vascular anatomy [8]. These advances provide the technical basis for more integrated cardiovascular MRA strategies.

Higher magnetic field strength may further facilitate such integrated imaging. At 5T, the increased intrinsic signal-to-noise ratio offers the potential for higher spatial resolution and improved vessel visualization, although greater B0/B1 field inhomogeneity and motion sensitivity remain important technical challenges [9]. Recent studies have demonstrated the feasibility of non-contrast coronary MRA at 5T. Lu et al. reported favorable coronary image quality and diagnostic performance using 5T non-contrast Dixon whole-heart coronary MRA [10]. More recent studies have further demonstrated improved coronary image quality and enhanced visualization of small coronary branches at 5T compared with 3T [11,12]. However, these investigations have primarily focused on the coronary arteries, and evidence remains limited regarding whether a single non-contrast 5T acquisition can provide adequate visualization of both the thoracic aorta and coronary arteries.

Heart rate (HR) represents an additional challenge for integrated cardiovascular MRA. Coronary image quality is strongly influenced by cardiac motion, and previous studies have demonstrated that higher HR is associated with shorter motion-free periods and reduced coronary image quality [13,14,15]. In contrast, the thoracic aorta has a substantially larger caliber and different motion characteristics, suggesting that the effect of HR may not be identical across the two vascular territories. The differential influence of HR on aortic and coronary image quality within the same large-coverage 5T acquisition has not been well characterized.

Therefore, this prospective study aimed to evaluate the feasibility of 5T non-contrast one-stop MRA for simultaneous visualization of the thoracic aorta and coronary arteries in healthy volunteers and to investigate the influence of HR on image quality across these two vascular territories.

## 2. Materials and Methods

### 2.1. Participants

This prospective study was approved by the Institutional Review Board of Beijing Anzhen Hospital (Approval Number: 2025232x), and written informed consent was obtained from all participants before the examination. From January to March 2025, thirty-four healthy adult volunteers were prospectively recruited for non-contrast, free-breathing 5T MRA. The inclusion criteria were (1) age ≥ 18 years and (2) no known history of cardiovascular disease. The exclusion criteria were (1) pregnancy; (2) the presence of an MRI-incompatible device or other contraindication to MRI; and (3) severe claustrophobia.

Clinical data, including age, sex, blood pressure, height, and body weight, were recorded before MRI. Body mass index (BMI) was calculated as weight divided by height squared (kg/m^2^). HR was recorded during the MRA examination. Based on the median HR (70 beats per minute [bpm]), participants were divided into two groups: a Low-HR group (≤70 bpm, *n* = 17) and a High-HR group (>70 bpm, *n* = 17). Clinical and imaging parameters were then compared between these two groups.

### 2.2. Image Acquisition

All examinations were performed on a 5T MRI system (uMR Jupiter; United Imaging Healthcare, Shanghai, China) using a 24-channel body coil. Electrocardiogram (ECG) gating was employed for cardiac synchronization. Participants were instructed to avoid caffeine and other stimulants for at least 24 h prior to the scan.

After obtaining localizer images, a free-breathing cine sequence was acquired in the four-chamber view to monitor the motion of the right coronary artery. The ECG trigger delay and cardiac acquisition window were individually determined for each participant according to the cardiac phase exhibiting minimal coronary motion on the cine images [6]. Therefore, the cardiac acquisition window was not fixed across participants. The respiratory navigator acceptance window was fixed at ±1 mm for all participants. Static radiofrequency (RF) shimming was performed during prescan calibration to improve B1+ homogeneity over the cardiac region. Transmit weights across multiple channels were optimized using a magnitude least-squares approach to reduce spatial variation in RF excitation associated with high-field cardiovascular imaging.

Both the thoracic aorta and coronary arteries were imaged simultaneously using an ECG-triggered, non-contrast-enhanced QUICK 3D Water Fat Imaging (QUICK3D_WFI) sequence [6]. AI-assisted compressed sensing (ACS) was used for acquisition acceleration, with an overall acceleration factor of 10.23. Key imaging parameters were as follows: field of view = 300 × 288 mm^2^, acquisition matrix = 300 × 288, voxel size = 1.0 × 1.0 × 1.2 mm^3^, flip angle = 10°, echo time = 1.85 ms, repetition time = 5.23 ms, slice thickness = 1.2 mm, phase oversampling = 50, and bandwidth = 1290 Hz.

### 2.3. Image Processing and Analysis

Image analysis was performed independently by two experienced radiologists (N.Z., with 15 years of experience in MRA interpretation, and R.Z., with 6 years of experience in MRA interpretation) at a post-processing workstation (uOmnispace.MR, United Imaging Healthcare Co., Ltd., Shanghai, China), both blinded to participant information. For intraobserver reproducibility assessment, N.Z. repeated the complete evaluation of all 34 datasets, including subjective image-quality scoring and objective measurements, after a 2-week interval. All final assessments, including subjective image-quality scores, SNR, CNR, and vessel edge sharpness (VES), were determined by averaging the evaluations of the two readers. This approach preserved the independent assessments of both readers while assigning equal weight to each reader in the final analysis, consistent with previous cardiovascular MRI studies [10,16,17,18].

The aorta was divided into four segments: the aortic sinus (Asin), ascending aorta (Asc A), aortic arch (Ao Arch), and descending aorta (Desc A). According to the Society of Cardiovascular Computed Tomography (SCCT) classification standards [19], the following nine coronary segments were evaluated: ① left main coronary artery (LM); ② proximal (LAD-pro), middle (LAD-mid), and distal (LAD-dis) segments of the left anterior descending artery (LAD); ③ proximal (LCX-pro) and distal (LCX-dis) segments of the left circumflex artery (LCX); and ④ proximal (RCA-pro), middle (RCA-mid), and distal (RCA-dis) segments of the RCA. The schematic diagram is shown in Figure 1.

#### 2.3.1. Subjective Image Quality Evaluation

All segments of the thoracic aorta and coronary arteries as defined above were subjectively evaluated. A four-point scale [7] was used to assess each segment as follows: 4: Vessel boundaries were sharply defined, with no motion artifacts; 3: Vessels were well visualized with mild artifacts; 2: Images contained substantial motion artifacts, but the vascular segment remained evaluable, albeit with significant limitations; 1: Vessels exhibited severe artifacts, rendering the segment non-evaluable. Scores ≥ 2 were considered evaluable for the purposes of this technical feasibility study [7].

#### 2.3.2. Objective Image Quality Evaluation

Objective image quality metrics—SNR, CNR, and VES—were evaluated for all aortic segments and the proximal segments of the major coronary arteries (LM, LAD-pro, LCX-pro, and RCA-pro).

The metrics were defined as follows:

- SNR: SI blood/SD noise;
- CNR: (SI blood − SI tissue)/SD noise;
- VES: computed using a gradient-based algorithm in MATLAB (R2019a; The MathWorks, Inc., Natick, MA, USA).

First, background noise (SDnoise) was estimated as the standard deviation of signal intensity within a background ROI. The background ROI was placed consistently at the same relative location, with a similar size across participants, while avoiding visible artifacts. Because spatially varying noise may occur with multichannel coils and accelerated MRI reconstruction, the resulting SNR and CNR values were interpreted as relative image-quality estimates rather than absolute measures of local SNR. Second, vessel lumen signal intensity (SI blood) was determined by placing a region of interest (ROI) centrally within the proximal portion of each vascular segment, maximizing the ROI area wherever possible to accurately measure blood signal intensity. Finally, perivascular tissue signal intensity (SI tissue) was measured by delineating an ROI within the perivascular adipose tissue surrounding both the aortic root and the LM coronary artery.

### 2.4. Statistical Analysis

Statistical analyses were performed using SPSS Statistics version 27.0 (IBM Corp., Armonk, NY, USA). The distribution of continuous variables was assessed using the Shapiro–Wilk test. Normally distributed continuous variables are presented as mean ± standard deviation (SD), whereas non-normally distributed variables are presented as median and interquartile range (IQR). Categorical variables are presented as numbers and percentages. Comparisons between the Low-HR and High-HR groups were performed using the independent-samples Student’s *t*-test for normally distributed continuous variables and the Mann–Whitney U test for non-normally distributed or ordinal variables. Categorical variables were compared using the chi-square test or Fisher’s exact test, as appropriate. To complement the group-based analysis, HR was additionally analyzed as a continuous variable using Spearman’s rank correlation. Multivariable linear regression analyses were further performed for the objective image-quality metrics (SNR, CNR, and VES), with adjustment for BMI. Interobserver and intraobserver agreement were assessed using weighted kappa statistics for subjective scores and intraclass correlation coefficients (ICC) for objective metrics. Given the exploratory nature of this technical feasibility study and the correlated image-quality measurements across vascular segments, no formal adjustment for multiple comparisons was applied. Therefore, segment-wise *p* values should be interpreted as exploratory. All statistical tests were two-sided, and a *p* value < 0.05 was considered statistically significant.

## 3. Results

### 3.1. Clinical Characteristics

A total of 34 participants were enrolled in the study, comprising 14 (41.2%) male and 20 (58.8%) female participants. The mean acquisition time was 11.4 ± 4.1 min. Both the Low-HR group and the High-HR group consisted of 17 subjects each. No significant differences were observed in baseline characteristics between the two groups. The baseline characteristics of the two groups are detailed in Table 1.

### 3.2. Feasibility Analysis

The 5T non-contrast one-stop MRA protocol was successfully performed in all 34 participants, providing complete coverage of both the thoracic aorta and coronary arteries in every case. All acquired vascular segments were evaluable (Figure 2), supporting the technical feasibility of the protocol. The protocol also demonstrated excellent reproducibility, with outstanding interobserver and intraobserver agreement for both subjective image quality (kappa ≥ 0.90) and objective quantitative measurements (ICC ≥ 0.85 for SNR, CNR, and VES across all evaluated segments).

### 3.3. Subjective Image Quality Score

Subjective image-quality scores indicated that all vascular segments in both the coronary and aortic regions were evaluable (score ≥ 2). However, significant intergroup differences were observed specifically in certain coronary segments between the Low-HR (≤70 bpm) and High-HR (>70 bpm) groups, while no such differences were found in aortic segments (Table 2 and Figure 3). For the thoracic aorta, scores were comparable between the two groups across all segments: aortic sinus (3.9 ± 0.3 vs. 3.6 ± 0.5, *p* = 0.20), ascending aorta (4.0 ± 0 vs. 3.9 ± 0.3, *p* = 0.15), aortic arch (3.9 ± 0.2 vs. 3.9 ± 0.2, *p* = 1.00), and descending aorta (both 4.0 ± 0, *p* = 1.00). In contrast, the Low-HR group demonstrated superior coronary artery scores in the LAD-dis (3.6 ± 0.6 vs. 3.0 ± 0.6; *p* = 0.01), LCX-pro (3.6 ± 0.6 vs. 3.2 ± 0.4; *p* = 0.03), LCX-dis (3.2 ± 0.7 vs. 2.6 ± 0.5; *p* = 0.03), and RCA-dis (3.1 ± 0.7 vs. 2.5 ± 0.6; *p* = 0.04).

### 3.4. Objective Image Quality Score

Objective image-quality metrics were consistently higher in the Low-HR group than in the High-HR group across all evaluated segments. SNR was significantly higher in all aortic segments for Low-HR versus High-HR subjects (e.g., aortic sinus: 67.0 ± 13.9 vs. 55.8 ± 11.4, *p* = 0.02; descending aorta: 75.0 ± 14.3 vs. 63.4 ± 13.4, *p* = 0.02) and coronary segments (e.g., LM: 78.1 ± 23.7 vs. 56.0 ± 10.0, *p* = 0.001). Similarly, CNR was elevated in the Low-HR group across aortic (e.g., ascending aorta: 65.9 ± 11.4 vs. 56.5 ± 9.1, *p* = 0.01) and coronary segments (e.g., RCA-pro: 77.1 ± 25.6 vs. 55.2 ± 14.3, *p* = 0.004). VES showed the most pronounced decline in High-HR subjects, particularly in the aortic sinus (172.6 ± 24.0 vs. 130.4 ± 6.4, *p* < 0.001) and RCA-pro (194.7 ± 14.3 vs. 170.2 ± 8.8, *p* < 0.001), with significant reductions in all measured segments (*p* < 0.05). The objective evaluation results of the images are presented in Table 3 and Figure 3. Figure 4 presents illustrative examples from one Low-HR participant and one High-HR participant. Additional analyses treating HR as a continuous variable showed a similar overall pattern. HR was negatively correlated with subjective image-quality scores in the LAD-dis, LCX-pro, and LCX-dis segments and with 20 of 24 objective image-quality measurements; after adjustment for BMI, significant negative associations remained for 15 of 24 objective measurements (Supplementary Tables S1 and S2). Representative continuous associations between HR and image-quality parameters are illustrated in Supplementary Figure S1.

## 4. Discussion

In this prospective study, we evaluated the feasibility of 5T non-contrast one-stop MRA for simultaneous visualization of the thoracic aorta and coronary arteries and investigated the influence of HR on image quality. The principal findings were as follows: First, the protocol was successfully completed in all 34 participants, and all evaluated aortic and coronary segments were evaluable based on subjective image quality assessment. Second, subjective image quality of the thoracic aorta remained comparable between the Low- and High-HR groups, whereas several coronary segments, particularly distal segments, showed lower subjective scores at higher HRs. Third, objective image-quality metrics, including SNR, CNR, and VES, were consistently higher in the Low-HR group across all quantitatively assessed aortic and proximal coronary segments. These findings demonstrate the technical feasibility of simultaneously evaluating the thoracic aorta and coronary arteries within a single non-contrast 5T acquisition, while also suggesting different sensitivities of the two vascular territories to HR variation.

Subjective image quality of the thoracic aorta appeared relatively insensitive to HR within the range evaluated in this study. No significant differences in subjective scores were observed between the Low- and High-HR groups across the four aortic segments, and all segments remained evaluable. Although SNR, CNR, and VES were significantly higher in the Low-HR group, these objective differences did not translate into significant differences in subjective aortic image quality. This may partly reflect the larger caliber and different motion characteristics of the thoracic aorta compared with the coronary arteries, making aortic visualization less susceptible to variations in HR. A ceiling effect of the four-point subjective scale may also have contributed to this discrepancy, because aortic image-quality scores were clustered near the upper end of the scale, potentially limiting the sensitivity of subjective assessment to modest differences detected by quantitative metrics. Previous studies have also demonstrated the feasibility and diagnostic image quality of non-contrast MRA for thoracic aortic assessment [4,5]. However, whether the relative stability observed in our study is maintained in patients with aortic disease requires further investigation.

In contrast, coronary image quality demonstrated a pronounced dependence on HR. Subjective scores for several distal coronary segments (LAD-dis, LCX-dis, and RCA-dis) and the proximal LCX were significantly lower in the High-HR group, while objective image-quality metrics in the proximal coronary segments were also lower in the High-HR group. This heightened sensitivity is physiologically grounded. Coronary imaging relies on the relatively motion-free diastolic window, which shortens as HR increases. This reduction in the effective acquisition time, combined with the smaller diameter and greater mobility of distal vessels, exacerbates motion artifacts and degrades image quality at higher HRs. This observation aligns with evidence from other imaging modalities. Studies in coronary CTA [14] and 3T non-contrast MRA [15] have similarly reported decreased image quality and diagnostic accuracy in distal coronary segments at elevated HRs. Although BMI and acquisition time showed modest between-group differences, BMI was accounted for in the adjusted analyses, whereas acquisition time was not treated as a baseline confounder because it is a scan-process variable measured during the same examination and may partly reflect the acquisition and cardiac-gating process.

Notably, despite the reduction in image quality at higher HRs, all evaluated coronary segments remained evaluable. The increased intrinsic SNR available at 5T may support high-spatial-resolution vascular imaging and facilitate visualization of small coronary structures [10,11]. In the present study, an acquired in-plane resolution of 1.0 × 1.0 mm^2^ was achieved, which may have contributed to maintaining coronary visualization even under less favorable HR conditions.

The 5T non-contrast MRA protocol was successfully implemented in all 34 volunteers. A key factor contributing to the feasibility of this protocol was the effective management of B1+ inhomogeneity at high field strength. High-field cardiac MRI is challenged by increased transmit B1 (B1+) inhomogeneity because of the shorter RF wavelength in human tissues, which can lead to spatial variations in flip angle and signal intensity [9]. To mitigate these effects, we implemented static RF shimming during prescan calibration [20,21]. By optimizing transmit weights across multiple channels using magnitude least squares optimization, we achieved improved B1+ homogeneity across the heart, particularly enhancing signal in regions such as the right ventricle, which is often poorly excited in conventional circularly polarized (CP) mode (Supplementary Figure S2) [22]. This technique reduces dielectric shading artifacts and ensures more consistent image quality, which is critical for reliable evaluation of both the aorta and coronary arteries in a single acquisition.

Furthermore, the implemented ECG-triggered, non-contrast QUICK3D_WFI sequence played a crucial role in achieving the high image quality observed in this study. Integrating a Gradient Recalled Echo with Spoiled Gradient Echo (GRE-SP)-based quick 3D scheme with water-fat separation, this sequence is characterized by a small flip angle and capabilities for rapid fat suppression and imaging. By rapidly acquiring multiple k-space lines (echo train length) after a preparatory fat-saturation or saturation band pulse, it achieves quick 3D acquisition, which contributes to reduced motion artifacts and enhanced final image quality. An additional advantage is its relative insensitivity to B_0_ field inhomogeneity, resulting in consistent fat suppression performance. These characteristics make it particularly beneficial for applications requiring large FOVs, imaging of regions with multiple cavities, and off-center examinations.

From a clinical perspective, this technique provides a potential radiation- and contrast-free approach for integrated cardiovascular assessment. The protocol enabled simultaneous evaluation of the thoracic aorta and coronary arteries with a mean acquisition time of 11.4 ± 4.1 min. However, the present findings should be interpreted primarily as evidence of technical feasibility rather than clinical diagnostic performance, given the small cohort of predominantly young healthy volunteers and the absence of comparison with a reference standard such as CTA or invasive coronary angiography. Therefore, its applicability to patients with cardiovascular disease and its diagnostic accuracy for vascular pathology require further clinical validation.

This study has several limitations. First, the sample size was relatively small and consisted predominantly of young, healthy volunteers, limiting generalizability to older individuals and patients with cardiovascular disease. Second, individuals with arrhythmias were not represented, and the performance of the protocol under irregular cardiac rhythms remains unknown. Third, coronary evaluation focused primarily on the major coronary arteries, and smaller side branches were not systematically assessed. Fourth, the multiple segment-wise comparisons were not adjusted for multiplicity; therefore, particularly marginal *p* values should be interpreted cautiously because of the potential risk of type I error. Fifth, SNR and CNR were estimated using background-noise SD, which may not fully account for spatially nonuniform noise associated with multichannel coils and ACS reconstruction; these measures should therefore be interpreted as relative rather than absolute image-quality estimates. Finally, no direct comparison with CTA, invasive coronary angiography, or conventional-field MRI was performed; therefore, the diagnostic accuracy of this 5T MRA protocol for coronary or aortic disease remains to be established in future clinical studies.

In conclusion, 5T non-contrast one-stop MRA is technically feasible for simultaneous visualization of the thoracic aorta and coronary arteries in healthy volunteers. Higher HR was associated with reduced coronary image quality, particularly in distal segments, whereas subjective aortic image quality remained relatively stable. These findings support further clinical validation of this radiation- and contrast-free cardiovascular imaging approach in patients with coronary and aortic disease.

## Figures and Tables

**Figure 1:**
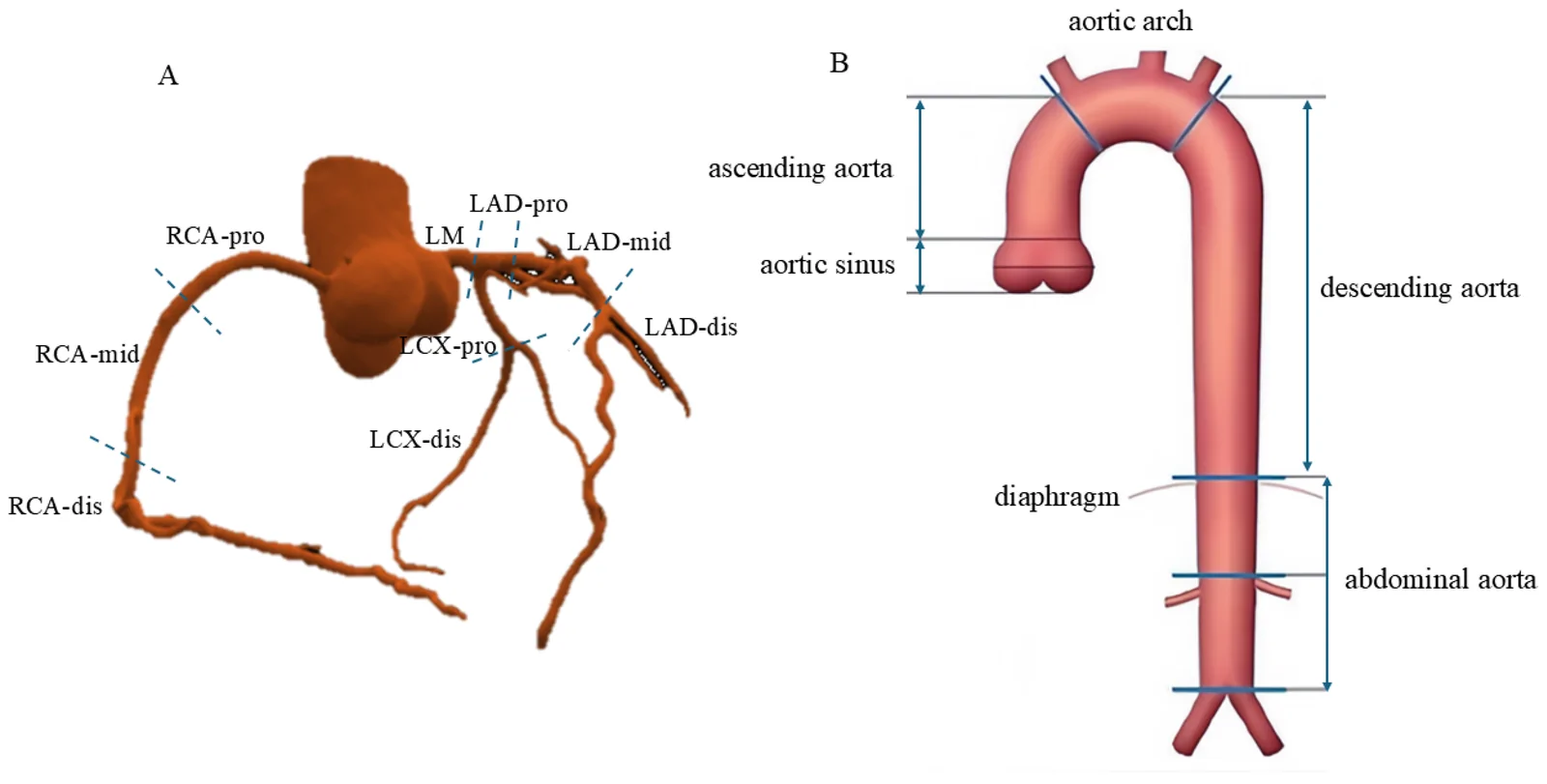
Segmental Anatomy of Coronary Arteries and Thoracic Aorta. Segmental anatomy of the three main coronary arteries according to the SCCT guidelines (**A**). Division of the thoracic aorta into four anatomical segments (**B**). Abbreviations: LM = left main coronary artery; LAD = left anterior descending coronary artery; LCX = left circumflex coronary artery; RCA = right coronary artery; pro = proximal segment; mid = middle segment; dis = distal segment.

**Figure 2:**
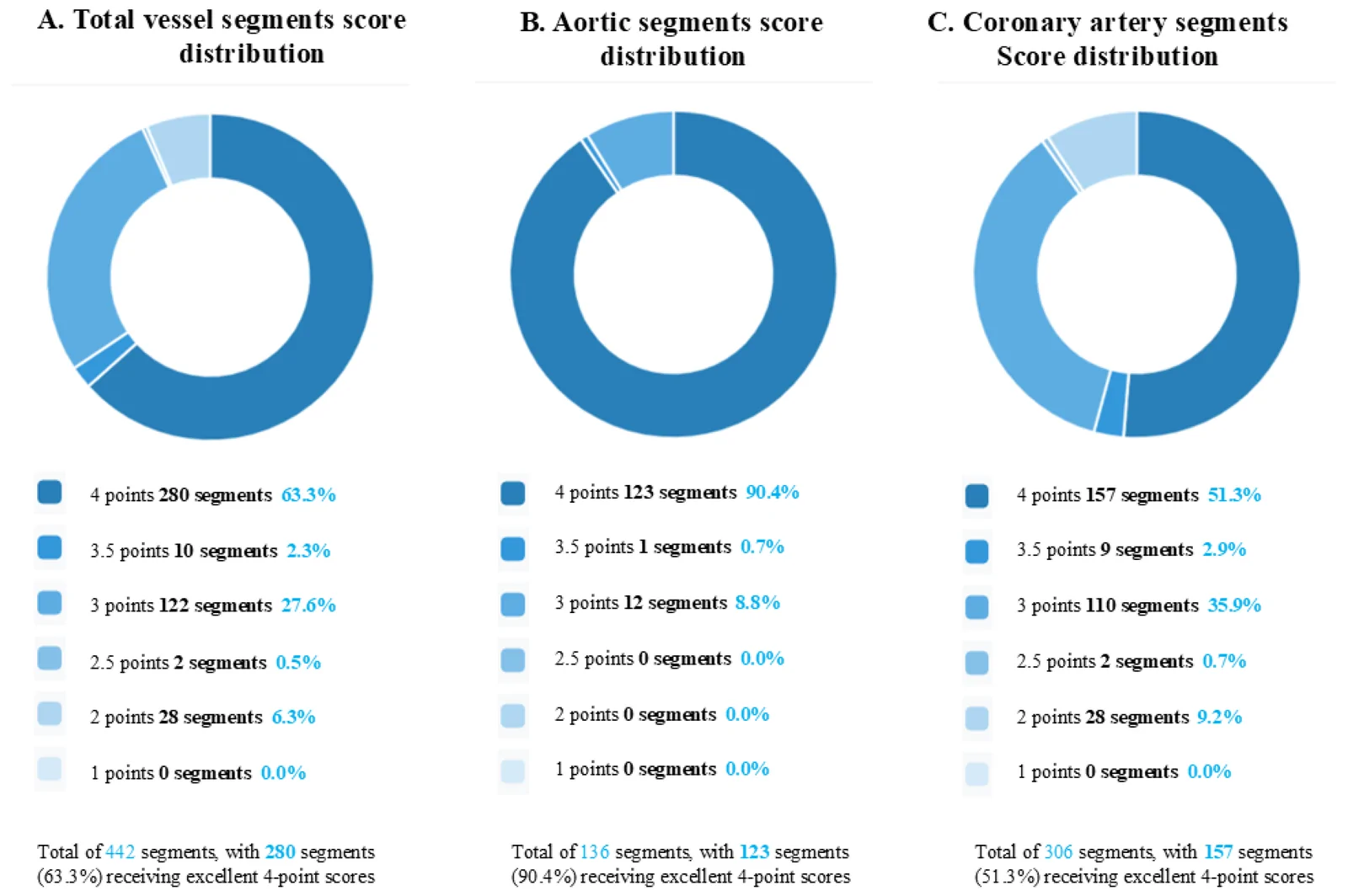
Overall subjective image quality scores across all participants: (**A**) Distribution of scores for all evaluated vascular segments combined. (**B**) Distribution of scores specifically for thoracic aortic segments. (**C**) Distribution of scores specifically for coronary artery segments. The subjective image quality was graded on a 4-point scale. The data labels within each bar indicate the number and percentage of segments achieving each score. All segments across both vascular territories were evaluable (score ≥ 2), supporting the high technical success rate of the protocol.

**Figure 3:**
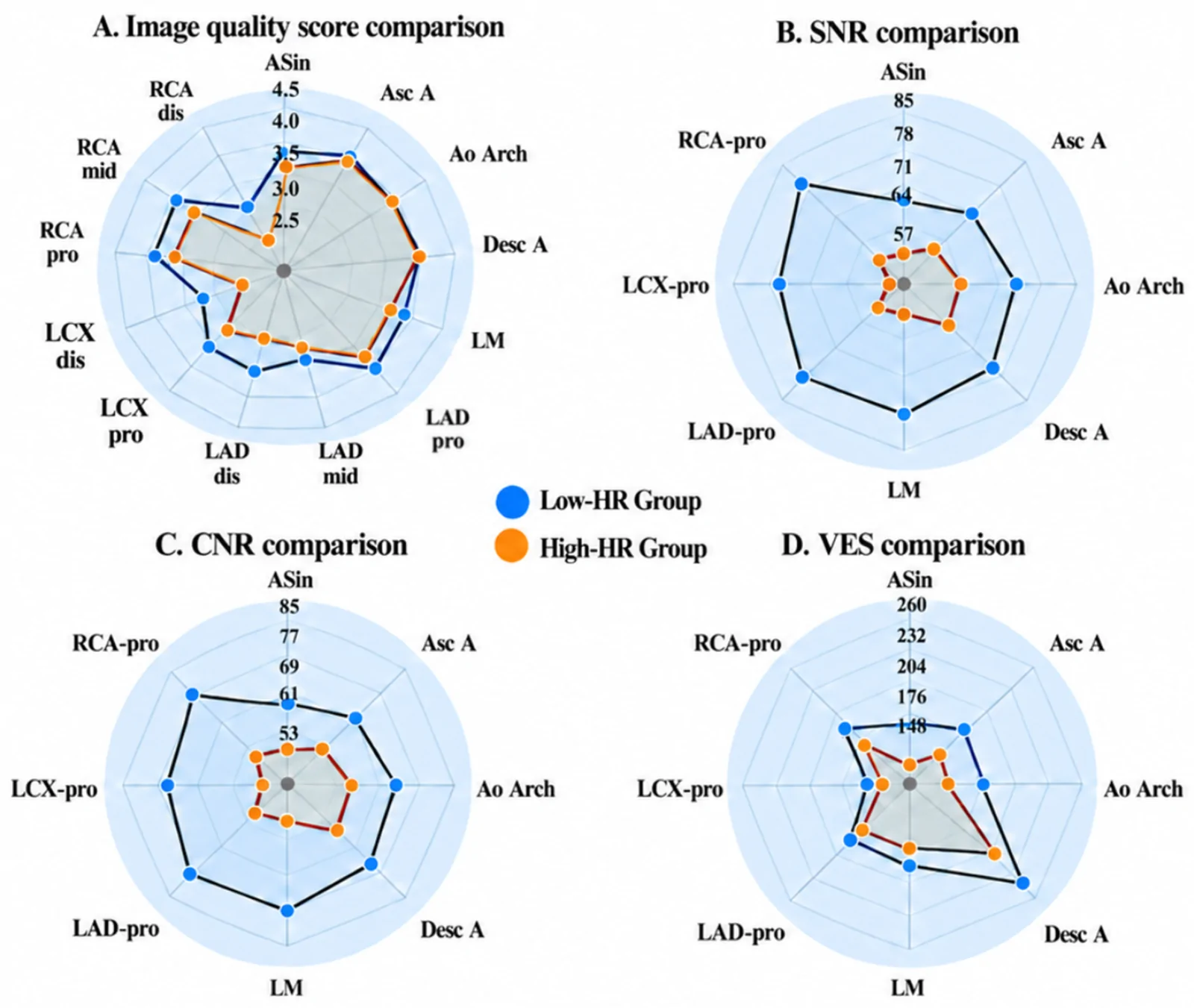
Comparison of subjective and objective image quality metrics between Low-HR and High-HR groups: (**A**) Subjective image-quality scores showed no significant differences in the aortic segments, whereas significant differences were observed in several coronary segments. (**B**–**D**) SNR, CNR, and VES differed significantly between the two groups at all evaluated anatomical sites (all *p* < 0.05).

**Figure 4:**
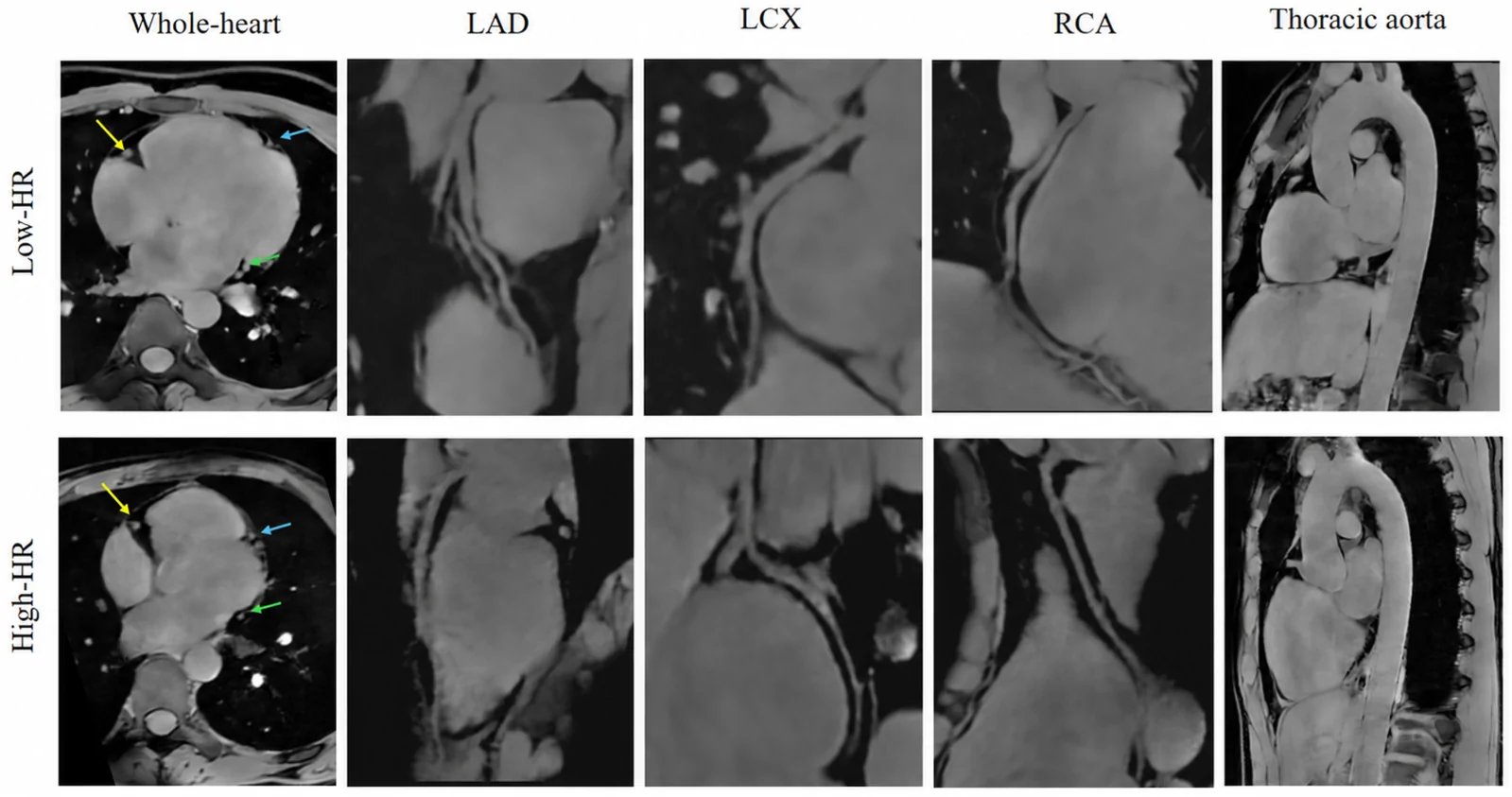
Representative images from a Low-HR subject and a High-HR subject. The Low-HR group (56 bpm) features a 28-year-old healthy volunteer; the High-HR group (72 bpm) shows a 26-year-old healthy volunteer. Axial whole-heart MR images demonstrate the RCA (yellow arrow), LAD (blue arrow), and LCX (green arrow), along with their respective reconstructions. Sagittal reconstruction visualizes the thoracic aorta. These images are provided for illustrative purposes only and should not be interpreted as demonstrating the overall differences between the Low- and High-HR groups.

**Table 1:** Baseline characteristics of the study population.

Parameter	Total *n* = 34	Low-HR Group *n* = 17	High-HR Group *n* = 17	*p* Value
Age (years)	24 (23, 25.3)	24 (23, 27)	24 (23.5, 25)	0.50
Male (*n*, %)	14 (41.2%)	5 (29.4%)	9 (52.9%)	0.30
BMI (kg/m^2^)	21.8 ± 3.0	20.6 ± 2.1	23.0 ± 3.4	0.08
HR (bpm)	70.3 ± 10.9	61.1 ± 4.5	79.5 ± 6.5	<0.001
SBP (mm Hg)	109.7 ± 9.8	106.5 ± 8.2	112.9 ± 10.4	0.15
DBP (mm Hg)	74.9 ± 7.4	73.9 ± 7.6	75.8 ± 7.3	0.79
Acquisition time (min)	11.4 ± 4.1	10.1 ± 3.7	12.7 ± 4.2	0.06

Abbreviations: BMI = body mass index; HR = heart rate; SBP = systolic blood pressure; DBP = diastolic blood pressure.

**Table 2:** Comparison of subjective image quality scores between Low- and High-HR groups.

Vascular Segment	Total *n* = 34	Low-HR Group *n* = 17	High-HR Group *n* = 17	*p* Value
Thoracic aorta				
Asin	3.8 ± 0.4	3.9 ± 0.3	3.6 ± 0.5	0.20
Asc A	3.9 ± 0.2	4.0 ± 0	3.9 ± 0.3	0.15
Ao Arch	3.9 ± 0.2	3.9 ± 0.2	3.9 ± 0.2	>0.99
Desc A	4.0 ± 0	4.0 ± 0	4.0 ± 0	>0.99
Coronary artery				
LM	3.8 ± 0.4	3.9 ± 0.3	3.7 ± 0.5	0.17
LAD-pro	3.9 ± 0.3	4.0 ± 0	3.8 ± 0.4	0.07
LAD-mid	3.3 ± 0.5	3.4 ± 0.5	3.2 ± 0.6	0.28
LAD-dis	3.3 ± 0.7	3.6 ± 0.6	3.0 ± 0.6	0.01
LCX-pro	3.4 ± 0.5	3.6 ± 0.6	3.2 ± 0.4	0.03
LCX-dis	2.9 ± 0.7	3.2 ± 0.7	2.6 ± 0.5	0.03
RCA-pro	3.8 ± 0.4	3.9 ± 0.3	3.6 ± 0.5	0.06
RCA-mid	3.8 ± 0.4	3.9 ± 0.3	3.6 ± 0.5	0.20
RCA-dis	2.8 ± 0.7	3.1 ± 0.7	2.5 ± 0.6	0.04

Abbreviations: Asin = aortic sinus; Asc A = ascending thoracic aorta; Ao Arch = aortic arch; Desc A = descending thoracic aorta; LM = left main coronary artery; LAD = left anterior descending coronary artery; LCX = left circumflex coronary artery; RCA = right coronary artery; pro = proximal segment; mid = middle segment; dis = distal segment.

**Table 3:** Comparison of objective image quality metrics between Low- and High-HR groups.

Parameter		Total *n* = 34	Low-HR Group *n* = 17	High-HR Group *n* = 17	*p* Value
SNR	Thoracic aorta				
	Asin	61.4 ± 13.7	67.0 ± 13.9	55.8 ± 11.4	0.02
	Asc A	63.4 ± 12.2	67.9 ± 12.8	59.0 ± 10.1	0.03
	Ao Arch	66.9 ± 13.6	71.5 ± 15.1	62.2 ± 10.4	0.046
	Desc A	69.2 ± 14.9	75.0 ± 14.3	63.4 ± 13.4	0.02
	Coronary artery				
	LM	67.1 ± 21.1	78.1 ± 23.7	56.0 ± 10.0	0.001
	LAD-pro	68.8 ± 21.9	79.7 ± 24.0	57.8 ± 12.7	0.002
	LCX-pro	63.2 ± 22.8	74.6 ± 25.8	51.9 ± 11.4	0.002
	RCA-pro	68.3 ± 24.7	80.0 ± 27.7	56.6 ± 14.2	0.004
CNR	Thoracic aorta				
	Asin	59.3 ± 12.9	64.2 ± 13.0	54.5 ± 11.2	0.03
	Asc A	61.2 ± 11.3	65.9 ± 11.4	56.5 ± 9.1	0.01
	Ao Arch	64.7 ± 13.7	69.9 ± 15.2	59.4 ± 9.9	0.02
	Desc A	67.1 ± 14.1	72.1 ± 13.7	62.2 ± 13.2	0.04
	Coronary artery				
	LM	65.0 ± 19.7	75.3 ± 21.7	54.7 ± 10.0	0.001
	LAD-pro	66.7 ± 20.5	76.9 ± 22.0	56.5 ± 12.6	0.002
	LCX-pro	61.1 ± 21.3	71.7 ± 23.8	50.5 ± 11.5	0.002
	RCA-pro	66.2 ± 23.2	77.1 ± 25.6	55.2 ± 14.3	0.004
VES	Thoracic aorta				
	Asin	151.5 ± 27.5	172.6 ± 24.0	130.4 ± 6.4	<0.001
	Asc A	168.9 ± 24.6	188.9 ± 15.6	149.0 ± 12.7	<0.001
	Ao Arch	156.8 ± 21.4	174.2 ± 14.6	139.4 ± 9.1	<0.001
	Desc A	227.6 ± 25.7	245.4 ± 15.1	209.7 ± 21.4	<0.001
	Coronary artery				
	LM	187.3 ± 15.7	195.5 ± 12.9	179.2 ± 14.2	0.001
	LAD-pro	182.8 ± 13.5	187.4 ± 11.8	178.2 ± 13.9	0.048
	LCX-pro	150.1 ± 10.9	153.8 ± 11.5	146.4 ± 9.1	0.04
	RCA-pro	182.4 ± 17.1	194.7 ± 14.3	170.2 ± 8.8	<0.001

Abbreviations: Asin = aortic sinus; Asc A = ascending thoracic aorta; Ao Arch = aortic arch; Desc A = descending thoracic aorta; LM = left main coronary artery; LAD = left anterior descending coronary artery; LCX = left circumflex coronary artery; RCA = right coronary artery; pro = proximal segment.

## Data Availability

The data presented in this study are available upon request from the corresponding author.

## Supplementary Materials

The following supporting information can be downloaded at: https://www.mdpi.com/article/10.3390/jcdd13100485/s1, Figure S1: Relationships between heart rate and representative image-quality parameters; Figure S2: B1+ Mapping of Cardiac at 5T; Table S1: Associations between continuous heart rate and image-quality parameters; Table S2: Multivariable associations between continuous heart rate and objective image-quality parameters.
