# Supplementary material for: 5T Non-Contrast One-Stop MRA for Thoracic Aorta and Coronary Arteries: A Prospective Feasibility Study in Healthy Volunteers
Source: J Cardiovasc Dev Dis. 2026 Sep 26;13(10):485. doi: 10.3390/jcdd13100485 (PMC13705245; doi:10.3390/jcdd13100485)
Supplement: Supplementary file 1 [file jcdd-13-00485-s001.zip › jcdd-4531518-supplementary.pdf]

Supplementary Table S1. Associations between continuous heart rate and image-quality parameters

Domain	Parameter	Segment	Spearman ρ	P value
Thoracic aorta	Subjective score	Asin	−0.229	0.193
Thoracic aorta	Subjective score	Asc A	−0.306	0.078
Thoracic aorta	Subjective score	Ao Arch	−0.115	0.518
Thoracic aorta	Subjective score	Desc A	—	—
Coronary artery	Subjective score	LM	−0.131	0.460
Coronary artery	Subjective score	LAD-pro	−0.270	0.123
Coronary artery	Subjective score	LAD-mid	−0.291	0.095
Coronary artery	Subjective score	LAD-dis	−0.429	**0.011**
Coronary artery	Subjective score	LCX-pro	−0.386	**0.024**
Coronary artery	Subjective score	LCX-dis	−0.354	**0.040**
Coronary artery	Subjective score	RCA-pro	−0.257	0.142
Coronary artery	Subjective score	RCA-mid	−0.173	0.327
Coronary artery	Subjective score	RCA-dis	−0.311	0.073
Thoracic aorta	SNR	Asin	−0.377	**0.028**
Thoracic aorta	SNR	Asc A	−0.326	0.060
Thoracic aorta	SNR	Ao Arch	−0.341	**0.048**
Thoracic aorta	SNR	Desc A	−0.363	**0.035**
Coronary artery	SNR	LM	−0.408	**0.017**
Coronary artery	SNR	LAD-pro	−0.420	**0.014**
Coronary artery	SNR	LCX-pro	−0.424	**0.013**
Coronary artery	SNR	RCA-pro	−0.381	**0.026**
Thoracic aorta	CNR	Asin	−0.352	**0.041**
Thoracic aorta	CNR	Asc A	−0.418	**0.014**
Thoracic aorta	CNR	Ao Arch	−0.391	**0.022**
Thoracic aorta	CNR	Desc A	−0.328	0.059
Coronary artery	CNR	LM	−0.368	**0.032**
Coronary artery	CNR	LAD-pro	−0.404	**0.018**
Coronary artery	CNR	LCX-pro	−0.429	**0.011**
Coronary artery	CNR	RCA-pro	−0.368	**0.032**
Thoracic aorta	VES	Asin	−0.756	**<0.001**
Thoracic aorta	VES	Asc A	−0.683	**<0.001**
Thoracic aorta	VES	Ao Arch	−0.780	**<0.001**
Thoracic aorta	VES	Desc A	−0.663	**<0.001**
Coronary artery	VES	LM	−0.457	**0.007**
Coronary artery	VES	LAD-pro	−0.304	0.081
Coronary artery	VES	LCX-pro	−0.330	0.057

Domain	Parameter	Segment	Spearman ρ	P value
Coronary artery	VES	RCA-pro	−0.666	**<0.001**

Note: N = 34. Spearman's rank correlation was used. Subjective image-quality scores are the mean of two independent reader ratings. For the descending aorta, the subjective score was constant across all participants, so ρ and P could not be estimated. These segment-wise analyses were exploratory; P values are unadjusted for multiple comparisons and should be interpreted accordingly.

Abbreviations: HR, heart rate; SNR, signal-to-noise ratio; CNR, contrast-to-noise ratio; VES, vessel edge sharpness; Asin = aortic sinus; Asc A = ascending thoracic aorta; Ao Arch = aortic arch; Desc A = descending thoracic aorta; LM, left main coronary artery; LAD, left anterior descending artery; LCX, left circumflex artery; RCA, right coronary artery; pro, proximal; mid, middle; dis, distal.

Supplementary Table S2. Multivariable associations between continuous heart rate and objective image-quality parameters

Domain	Metric	Segment	B for HR (95% CI)	Standardized β	P value	Adjusted R^2
Thoracic aorta	SNR	Aortic sinus	−0.463 (−0.912 to −0.014)	−0.367	**0.044**	0.087
Thoracic aorta	SNR	Ascending aorta	−0.293 (−0.701 to 0.115)	−0.260	0.154	0.050
Thoracic aorta	SNR	Aortic arch	−0.325 (−0.788 to 0.138)	−0.260	0.162	0.016
Thoracic aorta	SNR	Descending aorta	−0.472 (−0.957 to 0.013)	−0.345	0.056	0.093
Coronary artery	SNR	LM	−0.800 (−1.455 to −0.144)	−0.412	**0.018**	0.179
Coronary artery	SNR	LAD-pro	−0.793 (−1.484 to −0.103)	−0.393	**0.026**	0.156
Coronary artery	SNR	LCX-pro	−0.921 (−1.630 to −0.211)	−0.439	**0.013**	0.175
Coronary artery	SNR	RCA-pro	−0.866 (−1.659 to −0.073)	−0.381	**0.033**	0.125
Thoracic aorta	CNR	Aortic sinus	−0.382 (−0.812 to 0.049)	−0.321	0.080	0.056
Thoracic aorta	CNR	Ascending aorta	−0.312 (−0.681 to 0.058)	−0.301	0.095	0.082
Thoracic aorta	CNR	Aortic arch	−0.357 (−0.818 to 0.104)	−0.283	0.124	0.037
Thoracic aorta	CNR	Descending aorta	−0.382 (−0.851 to 0.087)	−0.294	0.107	0.064
Coronary artery	CNR	LM	−0.718 (−1.330 to −0.106)	−0.397	**0.023**	0.176
Coronary artery	CNR	LAD-pro	−0.711 (−1.358 to −0.064)	−0.377	**0.032**	0.151
Coronary artery	CNR	LCX-pro	−0.840 (−1.506 to −0.174)	−0.428	**0.015**	0.170
Coronary artery	CNR	RCA-pro	−0.784 (−1.531 to −0.036)	−0.367	**0.040**	0.119
Thoracic aorta	VES	Aortic sinus	−1.735 (−2.448 to −1.021)	−0.685	**<0.001**	0.429
Thoracic aorta	VES	Ascending aorta	−1.280 (−1.932 to −0.628)	−0.565	**<0.001**	0.404
Thoracic aorta	VES	Aortic arch	−1.500 (−1.956 to −1.043)	−0.763	**<0.001**	0.612
Thoracic aorta	VES	Descending aorta	−1.547 (−2.191 to −0.903)	−0.655	**<0.001**	0.465
Coronary artery	VES	LM	−0.580 (−1.070 to −0.090)	−0.401	**0.022**	0.172
Coronary artery	VES	LAD-pro	−0.345 (−0.805 to 0.114)	−0.277	0.136	0.020
Coronary artery	VES	LCX-pro	−0.295 (−0.657 to 0.066)	−0.295	0.106	0.062
Coronary artery	VES	RCA-pro	−0.917 (−1.377 to −0.457)	−0.583	**<0.001**	0.385

Note: N = 34. Separate multivariable linear regression models were fitted for each objective image-quality parameter. HR was entered as the variable of interest and BMI was included as the covariate. B represents the unstandardized change in the image-quality parameter per 1-beat/min increase in HR. These segment-wise analyses were exploratory; P values are unadjusted for multiple comparisons and should be interpreted accordingly.

Abbreviations: HR, heart rate; BMI, body mass index; CI, confidence interval; SNR, signal-to-noise ratio; CNR, contrast-to-noise ratio; VES, vessel edge sharpness; Asin = aortic sinus; Asc A = ascending thoracic aorta; Ao Arch = aortic arch; Desc A = descending thoracic aorta; LM, left main coronary artery; LAD, left anterior descending artery; LCX, left circumflex artery; RCA, right coronary artery; pro, proximal.

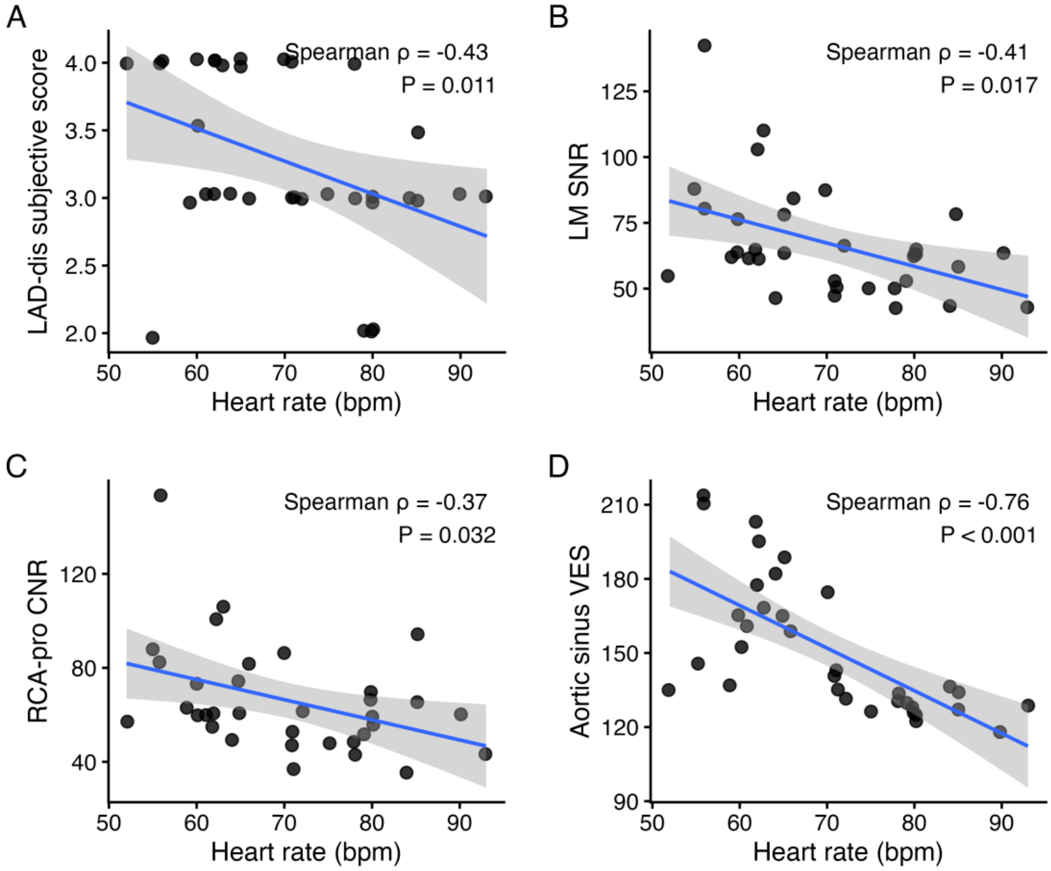

Supplementary Figure S1. Relationships between heart rate and representative image-quality parameters.

Scatter plots show the relationships between heart rate (HR) and representative subjective and objective image-quality parameters: (A) LAD-dis subjective image-quality score; (B) left main coronary artery (LM) signal-to-noise ratio (SNR); (C) proximal right coronary artery (RCA-pro) contrast-to-noise ratio (CNR); and (D) aortic sinus vessel edge sharpness (VES). Each point represents one participant. Solid lines and shaded areas represent fitted linear trends and their 95% confidence intervals for visualization. Spearman correlation coefficients (ρ) and corresponding P values are displayed in each panel.

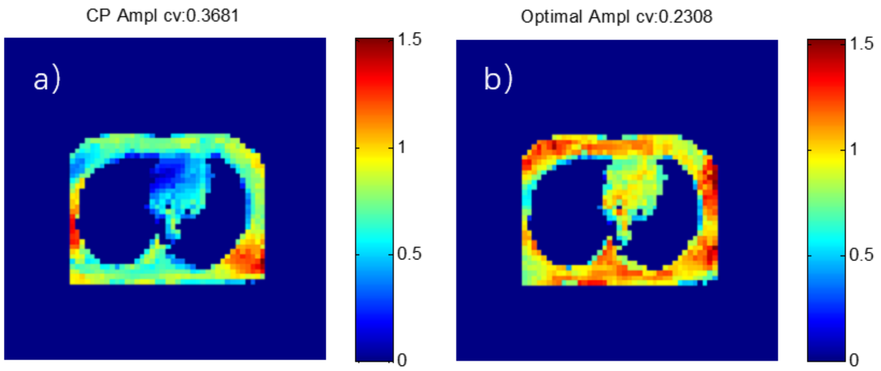

Supplementary Figure S2. B1+ Mapping of Cardiac at 5T a) B1+ sensitivity map of CP mode excitation. b) B1+ sensitivity map after static RF shimming

B1+ field distributions for the conventional CP mode and the optimized multi-transmit mode with static RF shimming in a four-chamber cardiac view. The shimmed case yielded a superior B1+ homogeneity, evidenced by a lower coefficient of variance (CV = 0.2308) compared to the CP mode (CV = 0.3681). Static RF shimming substantially improved the flip angle in the right ventricle, mitigating the severe signal dropout observed with the CP mode.
